# NPC1 is required for postnatal islet β cell differentiation by maintaining mitochondria turnover

**DOI:** 10.7150/thno.90946

**Published:** 2024-02-24

**Authors:** Bei Liu, Duanyi Hua, Linyan Shen, Tingting Li, Zheying Tao, Chenyang Fu, Zhongzheng Tang, Jie Yang, Li Zhang, Aifang Nie, Yiran Jiang, Jiqiu Wang, Yang Li, Yanyun Gu, Guang Ning

**Affiliations:** 1 Department of Endocrine and Metabolic Diseases, Shanghai Institute of Endocrine and Metabolic Diseases, Ruijin Hospital, Shanghai Jiao Tong University School of Medicine, Shanghai, China.; 2 Shanghai National Clinical Research Center for Metabolic Diseases, Key Laboratory for Endocrine and Metabolic Diseases of the National Health Commission of the PR China, Shanghai National Center for Translational Medicine, Ruijin Hospital, Shanghai Jiao Tong University School of Medicine, Shanghai, China.; 3 Department of Pharmacology, State Key Laboratory of Medical Neurobiology and MOE Frontiers Center for Brain Science, Key Laboratory of Metabolism and Molecular Medicine, Ministry of Education, School of Basic Medical Science, Fudan University, Shanghai, China.

**Keywords:** NPC1, β cell differentiation, diabetes, lysosome, mitophagy

## Abstract

**Rationale:** NPC1 is a protein localized on the lysosome membrane regulating intracellular cholesterol transportation and maintaining normal lysosome function. GWAS studies have found that *NPC1* variants in T2D was a pancreatic islet expression quantitative trait locus, suggesting a potential role of NPC1 in T2D islet pathophysiology.

**Methods:** Two-week-old *Npc1*^-/-^ mice and wild type littermates were employed to examine pancreatic β cell morphology and functional changes induced by loss of *Npc1*. Single cell RNA sequencing was conducted on primary islets. *Npc1*^-/-^ Min6 cell line was generated using CRISPR/Cas9 gene editing. Seahorse XF24 was used to analyze primary islet and Min6 cell mitochondria respiration. Ultra-high-resolution cell imaging with Lattice SIM^2^ and electron microscope imaging were used to observe mitochondria and lysosome in primary islet β and Min6 cells. Mitophagy Dye and mt-Keima were used to measure β cell mitophagy.

**Results:** In *Npc1*^-/-^ mice, we found that β cell survival and pancreatic β cell mass expansion as well as islet glucose induced insulin secretion in 2-week-old mice were reduced. *Npc1* loss retarded postnatal β cell differentiation and growth as well as impaired mitochondria oxidative phosphorylation (OXPHOS) function to increase mitochondrial superoxide production, which might be attributed to impaired autophagy flux particularly mitochondria autophagy (mitophagy) induced by dysfunctional lysosome in *Npc1* null β cells.

**Conclusion:** Our study revealed that NPC1 played an important role in maintaining normal lysosome function and mitochondria turnover, which ensured establishment of sufficient mitochondria OXPHOS for islet β cells differentiation and maturation.

## Introduction

Niemann-Pick disease type C intracellular cholesterol transporter 1 (NPC1) is a protein localized on the lysosome membrane that predominantly function as a transporter to escort cholesterol out of the lysosome 1. Mutated and loss of function of this gene lead to cholesterol accumulation in the lysosome, impairing lysosomal function and developing an autosomal recessive lysosomal storage disorder (LSD): Niemann-Pick disease type C1. Major defects of this disease manifest as nervous system disorders and hepatosplenomegaly 2, 3. GWAS studies have reported that the SNPs in *NPC1* are significantly associated with obesity and Type 2 diabetes mellitus (T2D) 4. Moreover, heterozygous mice with *Npc1* gene deletion are more likely to develop obesity and glucose intolerance phenotypes fed on a high fat diet 5, 6. Recently, the TIGER study has analyzed a large multi-cohort dataset of pancreatic islets with gene expression and dense genotyping data and found a common *NPC1* variant as an expression quantitative trait locus (eQTL) that decreases islet *NPC1* expression with increased T2D risk 7. This new evidence not only verifies the role of NPC1 in T2D pathogenesis but also emphasizes that NPC1 could be key to maintain normal human β cell functional repertoire, which has not yet been investigated.

Lysosomes have long been considered as an organelle that predominantly function as an intracellular trash bag to degrade either exogenous pathogens, wastes, or endogenous disabled organelles. Lysosome role as a hub for intracellular metabolism, signaling and quantity control has been increasingly recognized 8. Plenty of evidence has suggested that lysosome dysfunction is one of the pathogeneses of T2D 9, 10. One of the major defects caused by lysosomal dysfunction in cells, was autophagy blockage. Autophagy maintains cell metabolism homeostasis, including the quantity and quality control of other cell organelles. Evidences have shown that both HFD and aging suppress β cell autophagy, which elicits multiple disorders to promote β cell failure 2, 11, 12. However, whether perturbed autophagy induced by *Npc1* loss might exert negative effect on β cell maturation awaits to be explored.

Despite being important for adult β cell functional mass, few studies have examined the role of lysosome or autophagy in β cell development, maturation or differentiation in early life. Postnatal β cell maturation requires increasing glucose but decreasing amino acid induced mitochondria oxidative phosphorylation (OXPHOS) 13, 14. Switching of AMPK and mTOR signaling underlies above metabolic reprogramming induced by diet switch upon weaning and drives the cell maturation progress. As a hub to coordinate the balance between AMPK and mTOR signals and a main intracellular organelle to sense nutrient availability 15, lysosome in β cell is theoretically important for cell differentiation or maturation process during weaning when feeding pattern is substantially switched. Moreover, as the key organelle that assists β cell maturation, mitochondria also rely on lysosome activity to undergo sufficient mitophagy to achieve mitochondria quantity control and function homeostasis 16-18. However, there is lacking of direct evidence to evaluate the role of lysosome or mitophagy in postnatal β cell differentiation during weaning.

Herein, we used neonatal pups of *Npc1* knockout mice (*Npc1*^-/-^) and *Npc1* knockout Min6 cells (*Npc1*^-/-^ Min6 cell) to study the effect of *Npc1* deletion on β cell and found that *Npc1* loss induced the accumulation of autophagosome/lysosomes, decreased lysosome function, blocked cell autophagy, i.e., mitophagy, and hence impaired mitochondrial OXPHOS to perturb the normal postnatal differentiation of pancreatic β cells.

## Methods

### Animals

C57BL/6J genetic background *Npc1*^-/-^ mice were generated by mating *Npc1*^+/-^ mice 19. Heterozygous male and female mice were mated to produce homozygous mice and wild type littermates. Genotyping primer sequence: WT, Forward: 5'-GGTGCTGGACAGCCAAGTA-3', Reverse: 5'-TCTCACAGCCACAAGCTTCC-3'; MUT, Forward: 5'-TGAGCCCAAGCATAACTTCC-3', Reverse: 5'-GGTGCTGGACAGCCAAGTA-3'. All mice were housed in SPF facility with 12 h light/12 h dark cycle at steady room temperature (22-24 °C) with free access to water and food. All animal protocols were approved by the Animal Care Committee of Shanghai Jiao Tong University School of Medicine.

### Cell culture

Min6 cells were cultivated in DMEM (Thermo Fisher Scientific, Waltham, MA, USA) including 25 mM glucose, 15% FBS, 2 mM L-glutamine, 1 μl/mL β-mercaptoethanol and 1x ZellShield (Minerva Biolabs, Berlin, Germany) at 37 °C in 5% CO_2_ incubators. Bafilomycin A1 (BafA1, 20 nM, 24 h) and carbonyl cyanide phenylhydrazone (CCCP, 1 μM, 3 h) were used to treat Min6 cells.

### Isolation, Culture and Dissociation of Mouse Pancreatic Islets

The method of isolate primary mouse islets was described in our previous study 4. Collagenase P was used for pancreatic digestion (Roche Applied Science, Basal, Switzerland). *In vitro*, primary islets were cultured in medium containing RPMI 1640 with 10% FBS, 2 mM L-glutamine, 1% HEPES, 1x ZellShield (Minerva Biolabs, Berlin, Germany) at 37 °C in 5% CO_2_ incubators. The islet dissociation was performed with 0.25% Trypsin-EDTA, at 37 ℃ for 2min in 5% CO_2_ incubators.

### Glucose-Stimulated Insulin Secretion

The primary islets were isolated and handpicked. After incubation with culture medium mentioned above for 4 h, 20 islets/well were hand-picked in KRBB (4.8 KCl, 25 NaHCO_3_, 20 NaCl, 1.2 KH_2_PO_4_, 1.2 MgSO_4_, 2.5 CaCl_2_, 10 HEPES, in mM) for equilibrium with 2.8 mM glucose 1 h, then replace with fresh KRBB with 2.8 mM and 16.7 mM glucose separately. The supernatants were collected after glucose stimulation and islets were lysed in hydrochloric ethanol. The insulin levels in the supernatants and islet lysates were analyzed using a Mouse Insulin ELISA Kit (ALPCO, Salem, NH, USA) according to the instruction of manufacturer.

### qRT-PCR Analysis

Total RNA was extracted from islets or Min6 cells by TRIzol reagent (Thermo Fisher Scientific) and used for reverse transcription to cDNA using a PrimeScript RT reagent Kit (TaKaRa, Shiga, Japan). Gene expression was analyzed by quantitative real-time PCR of cDNA using SYBR Green (TaKaRa) on a LightCycler 480 (Applied Biosystems, Waltham, MA, USA). All β cells qRT-PCR data were normalized to *Actb* or *Gapdh*. Primers are listed in Table S1.

### Western Blot and CTSB activity

Total protein extracted from islets or Min6 cells was quantified using BCA Protein Assay Kit (Thermo Fisher Scientific). Proteins were separated by electrophoresis and transferred onto a PVDF membrane. Primary antibodies are listed below: anti-LAMP1 (1:1000, Abcam, Cambridge, U.K), anti-NPC1 (1:1000, Novus Centennial, CO, USA), anti-ATG5 (1:1000, Cell Signaling Technology, Danvers, MA, USA), anti-CTSB (1:1000, Cell Signaling Technology), anti-LC3 (1:1000, Cell Signaling Technology), anti-p62 (1:1000, Cell Signaling Technology), anti-pS6 (1:1000, Cell Signaling Technology), anti-p-AMPK (1:1000, Cell Signaling Technology), anti-COX4 (1:1000, CST), anti-OXPHOS (1:1000, ABCAM), anti-CHOP (1:1000, CST), anti-OPA1 (1:1000, CST), anti-PINK1 (1:1000, NOVUS), anti-ALDH1A3 (1:1000, NOVUS), anti-p-eIF2α (1:1000, CST), anti-eIF2α (1:1000, CST), anti-Caspase-3 (1:1000, Cell Signaling Technology), anti-Cleaved Caspase-3 (1:1000, Cell Signaling Technology), anti-β-Actin (1:10000, Aksomics), anti-HSP90 (1:10000, Santa Cruz Biotechnology).

CTSB activity was measured using a Cathepsin B Activity Assay Kit (Abcam) according to manufacturer's instruction. Briefly, reaction buffer 50 μL and 10 mM Cathepsin B substrate 2 μL were incorporated to protein lysates from Min6 cells (1x10^^^6) and incubated for 30 min at 37 °C before being read with 400 nm excitation and 505 nm emission. Activities were normalized to the CTSB protein levels of each sample.

### Immunochemistry and immunofluorescence

The pancreas was weighed, fixed, processed, and embedded in paraffin. Whole pancreas was then sectioned with 5 µm thickness, then sections were selected at intervals of 250-300 µm for immunohistochemical staining of insulin and counterstained with eosin. The sections were then scanned using an Axio Scan.Z1 (ZEISS, Oberkochen, Germany). The proportion of insulin-positive in total pancreatic staining area versus the whole pancreas area was calculated using image J. Immunofluorescence staining images were captured by confocal microscope LSM 880 (ZEISS). Antibodies used in the study included anti-Insulin (1:800, DAKO, Copenhagen, Denmark), anti-UCN3 (1:500, Phoenix Pharmaceuticals, Mannheim, Baden-Wurttemberg, Germany), anti-Ki67 (1:400, DSHB), anti-Glucagon (1:500, Abcam), anti-Somatostatin (1:100, Abcam), and anti-ALDH1A3 (1:100, NOVUS). Fluorescence detection was operated with Alexa Fluor 647 nm, 488 nm and 594 nm conjugated antibodies (Thermo Fisher Scientific). Cell nuclei were labelled with DAPI.

### Filipin Staining

Mouse pancreases were dehydrated with sucrose and frozen for 7 µm section. Then permeabilization was performed using 0.2% Triton and incubated with 50 µg/µL filipin (Sigma, St. Louis, MO, USA) in PBS for 45 min. Imaging was taken using a confocal microscope LSM880 (ZEISS).

### Ultrastructure observation with Electron Microscopy (EM)

The pancreatic tissue containing the islets was removed under stereomicroscope (Leica, Wetzlar, Germany), fixed, dehydrated, stained and embedded as previously reported 20. EM images were taken by Transmission Electron Microscope (HITACHI, Japan). Maturation of insulin vesicles was measured and calculated by 5000-fold magnification and representative images were collected at 20000-fold magnification by Image J.

### Lysosome digestive function and pH test

Min6 cells were cultured in 35 mm glass bottom dish or 12-well plates. Lysosome digestive function was detected using dye quenched bovine serum albumin (DQ-BSA-488 nm, Sharebio, Shanghai, China) and Lysosomal pH was detected using LysoSensor (Thermo Fisher Scientific, Waltham, MA, USA). Cells were incubated with DQ-BSA (10 µg/mL) for 6 h or LysoSensor (0.1 mg/mL) for 5 h at 37 ℃. Images were taken with confocal LSM880 (ZEISS). For lysosome digest function, fluorescence intensity at 488 nm excitation was measured. For lysosome pH, R/G ratio: the ratio of excitation 335 nm emission 452 nm (green, G) and excitation 381 nm emission 521 nm (red, R) was calculated to indicate the lysosome pH level. Fluorescent intensity quantification using Cytation5 (BioTek, Winooski, VT, USA).

### Mt-Keima test and Mitophagy dye

Min6 cells were cultured in a 35 mm glass bottom dish. Adenovirus carried with mt-Keima 21 plasmid (HG-VYA1474, Addgene) was incubated in Min6 and *Npc1*^-/-^cells. Imaging was taken using confocal LSM880 (ZEISS), GFP excitation was at 458 nm and RFP at 594 nm, emission both were at 626 nm, fluorescence intensity of each excitation was quantified using the Image J Software. Mitophagy level is calculated by excitation fluorescence of RFP versus (RFP+GFP) 22. Min6 cells were cultured in a 35 mm glass bottom dish. Mitophagy activity was detected using a Mitophagy Detection Kit (Dojindo, Kumamoto, Japan) 23, 24. Cells were first incubated with Mitophagy dye (100 nM) for 30 min, then with Lyso dye (1 μM) for another 30 min. Imaging was taken using confocal LSM880 (ZEISS). Fluorescence intensity was quantified using the Image J Software.

### MtDNA Content

Min6 cells DNA was extracted using Universal Genomic DNA Purification Mini Spin Kit (Beyotime). MtDNA Content was represented with the ratio of relative abundancy of mitochondrial DNA and genomic DNA by PCR amplification primer sequence: mCОX2 mitochondria DNA, Forward: 5'-CCCGAОTAAATОAACCAACA-3', Reverse: 5'-CAATGGCCATAAACCTATGC-3'. mβ-globin genomic DNA, Forward: 5'-GAASCGATTCIACGGAGCAG-3', Reverse: 5'-GGACCAGCGATICTCAGTACA-3'.

### Ultrahigh Resolution Cell Imaging

Min6 cells were cultured in a 35 mm glass bottom dish. Mitochondria were labeled with MitoTracker Green 1 μM (Cell Signaling Technology), Lysosome with Lysotracker Red 1 μM (Thermo Fisher Scientific), Nucleus with Hoechst (Beyotime), and incubated for 10 min at room temperature. Ultrahigh resolution imaging was conducted using Ultra-high-resolution Lattice SIM^2^(ZEISS).

### Autophagosome and mitochondria Imaging

Min6 cells were cultured in a 35 mm glass bottom dish. Autophagosomes were labeled by adenovirus infection which carried with LC3-mCherry sequence 25, mitochondria were labeled with MitoTracker Green 1 μM (Cell Signaling Technology). Imaging was taken using confocal LSM880 (ZEISS).

### CRISPR/Cas9 mediated *Npc1* knockout in Min6 cells

To obtain* Npc1* knockout Min6 cells, guide RNA targeting exon6 were designed and selected through http://crispor.tefor.net: gRNA-A1: CAGGACTGCTCCATCGTCTGCGG, gRNA-A2: CATCATGTGGGTCACCTACGTGG 26. Plasmids containing CRISPR-Cas9 RNP (Haixing Bioscience, Jiangsu, China) expression cassettes for hSpCas9 and chimeric gRNAs were electro-transfected by Neon transfection system according to the protocols of manufacturer (Thermo Fisher Scientific). After two days, single colonies were transferred into 96-well plates. To determine the presence of deletions in targeted clones, genomic DNA was isolated using a Quick-DNA Miniprep kit (Zymo Research, Irvine, CA, USA) and PCR amplification was achieved using 2×Taq Master Mix (Vazyme, Jiangsu, China) of primers flanking exon; Forward: 5'-ACAACGGACAAGCGCCATTTA-3'; Reverse: 5'-CAGATCCTCCAGGGCATAGGA-3'. Clones with mutations in both alleles were selected for downstream studies and maintained under the same conditions as parental cells.

### Oxygen Consumption Rate (OCR) and Mitochondrial superoxide (MitoSOX) Measurement

Islet OCR was measured using a Seahorse XF24 extracellular flux analyzer (Seahorse Bioscience, MA, USA). Each well was preincubated with 40-60 islets or 4 x 10^4^ cells in assay medium with 0.2% BSA and 2.8 mM glucose for 4 h for preparation, then 20 mM Glucose, 5 μM oligomycin, 4 μM FCCP and rotenone 5 μM (for cell: 2 μM oligomycin, 2 μM FCCP and 0.5 μM rotenone) were incorporated sequentially to measure mitochondrial OCR parameters (the above reagent is the final concentration).

Mitochondrial superoxide level was labeled using MitoSOX Red Mitochondrial superoxide indicator (M36008, Molecular Probes, USA), and analyzed by flow cytometry according to the manufacturer's protocol.

### Electrophysiology

A patch clamp was performed in the standard whole-cell configuration with the Sine+DC LockIn function of an EPC 10 amplifier and PatchMaster Software (HEKA Electronik, Lambrecht, Germany) 5-30 min after switching cells to the bath solution. Cells were preincubated in DMEM with 10% FBS, 100 units/mL penicillin/streptomycin, and 2.8 mM glucose for 1 h prior to switching to bath solution. Bath solution containing (in mM): 118 NaCl, 20 TEA, 5.6 KCl, 1.2 MgCl_2_, 2.6 CaCl_2_, 5 HEPES, and with glucose as indicated (pH adjusted to 7.4 with NaOH) in a heated chamber (32-35 ℃). The pipette solution contained (in mM): 125 Cs-glutamate, 10 CsCl, 10 NaCl, 1 MgCl_2_, 0.05 EGTA, 5 HEPES, cAMP and 3 MgATP (pH adjusted to 7.15 with CsOH). Exocytotic responses were measured 1-2 min after obtaining the whole-cell configuration in response to ten 500-ms depolarizations to 0 mV from a holding potential of -70 mV. Changes in capacitance were normalized to cell size (fF/pF).

### Single-cell Sequencing

After isolation and extraction, each tube contained 200-300 islets; they were digested with 0.25% Trypsin-EDTA, at 37 ℃ for 2 min. Digestion was stopped using RPMI 1640 (Thermo Fisher Scientific) containing 10% fetal bovine serum (FBS) (Thermo Fisher Scientific), blowing evenly. It was centrifuged at 1360 rpm for 5 min at 4 ℃. After the supernatant was abandoned, it was resuspended with 4 ℃ PBS containing 0.2% BSA, with cell concentration control at approximately 1000 counts/μL. Cell viability was measured with trypan blue staining and counted using a Cell Counter. Approximately 10,000-15,000 dissociation islet cells were operated using 3' library chips on the Chromium Single Cell 3' Library (v2) according to 10X Genomics platform. Gel Bead-In-Emulsions (GEMs) were formed by the microfluidization of each cell, and cell lysis, reverse transcription reaction and amplification were carried out within GEMs. The sequencing strategy used was the Illumina NovaSeq PE150. Data analysis (merge samples) using Cellranger, normalized parameter mapping. Cell filtration was performed with Seurat v4.0.3, removing low quality sequencing data (parameters: nFeature > 1000, nCount < 50000, mt < 10, percent of rbc > 0.1, double cells). Standardized method: SCTransform. Cell clustering was done using the FindClusters method with variable-feature 3000 and resolution 0.46. Differentially expressed genes (DEGs) analysis was operated with FindMarkers, |avg_logFC| > 0, p_val_adj < 0.01. Kyoto Encyclopedia of Genes and Genomes (KEGG) analysis was performed using David (https://david.ncifcrf.gov/).

### Statistical Analysis

The data were presented using Mean ± SEM, using a two-tailed student t test for two groups, and analyzed by GraphPad Prism 8.3.0 (GraphPad Software, USA). *P* < 0.05 was considered statistically significant.

## Results

### *Npc1* knockout reduced β cell mass in mice

**Figure 1:**
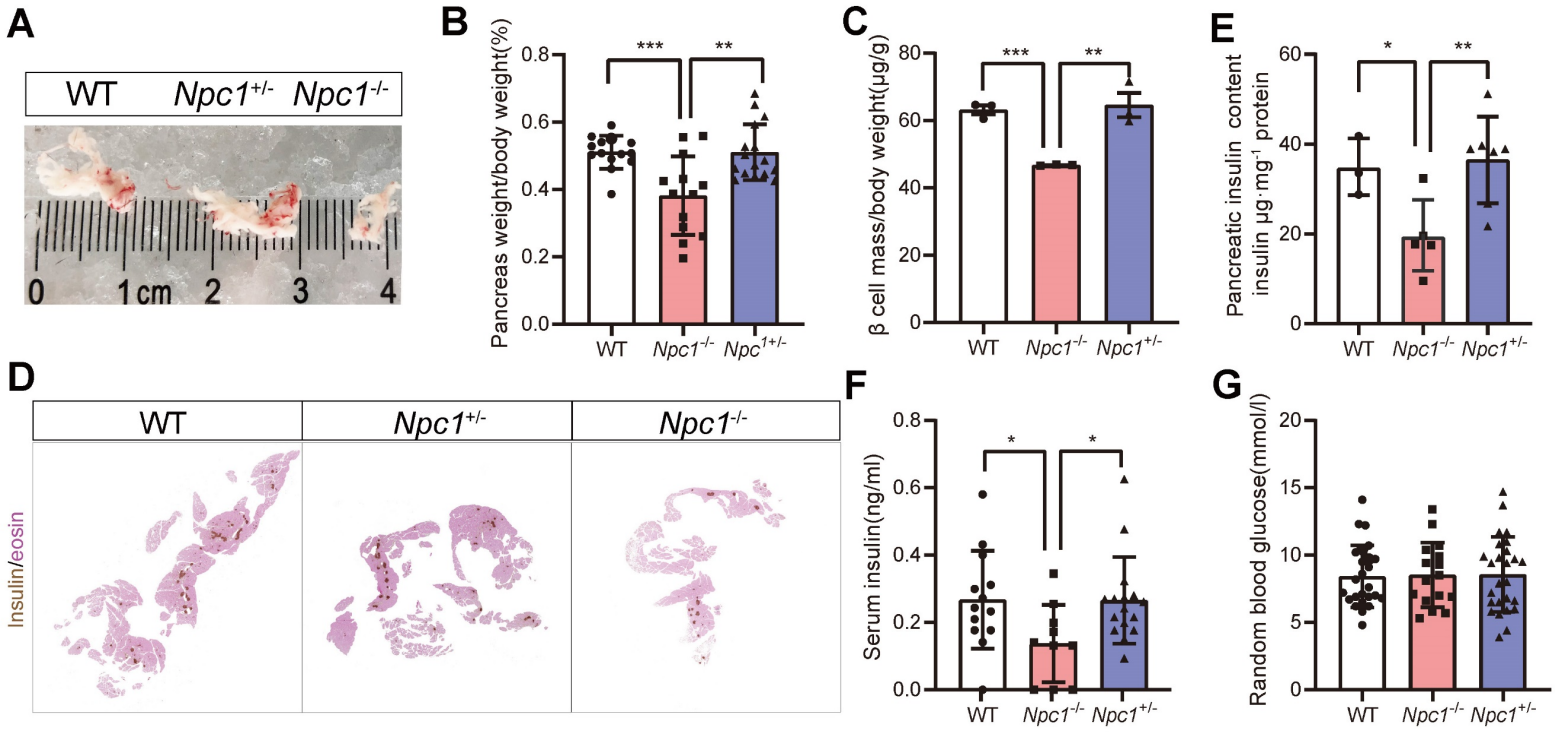
***Npc1* knockout reduced β cell mass in 2-week-old mice. A**. Representative images of whole pancreas dissected from 2-week-old mice. **B**. Pancreas weight versus body weight (n = 14-16). **C**. β cell mass normalized to body weight (n = 3). **D**. Representative images of immunochemistry staining of insulin with eosin counterstaining on continuous sections of pancreas in 2-week-old WT, *Npc1*^-/-^, and *Npc1*^+/-^ mice. **E**. Pancreas insulin content in 2-week-old WT, *Npc1*^-/-^, and *Npc1*^+/-^ mice (n = 3-7).** F**. Serum insulin level in 2-week-old WT, *Npc1*^-/-^, and *Npc1*^+/-^mice (n = 10-16). **G**. Random blood glucose levels in 2-week-old WT, *Npc1*^-/-^, and *Npc1*^+/-^ mice (n = 16-25). Data are presented as the mean ± S.E.M, * P < 0.05, ** *P* < 0.01, *** *P* < 0.001. Unpaired two-tailed Student's t test.

Consistent with a previous report 27, *Npc1*^-/-^ mice have a shorter life span (Figure S1A), and significant growth retardation compared to their wild-type (WT) and heterozygous (HET) littermates, with lower body weight, shorter body length (Figure S1B-C) and lower blood total cholesterol level (Figure S1D). We then examined phenotypes in mice with 2 weeks of age, before the decease of *Npc1^-^*^/-^ mice. The proportion of organ weight to body weight like brain and liver, increased in *Npc1*^-/-^ mice (Figure S1E-H). However, the organ size and proportion of pancreas weight in *Npc1*^-/-^ mice were substantially smaller than those in mice of other genotypes (Figure 1A-B). Meanwhile, the endocrine pancreas was also affected, after adjusted with whole pancreas weight, β cell mass were still significantly reduced in *Npc1*^-/-^ mice (Figure 1C-D), so was pancreas insulin content (Figure 1E), though with no changes in proportion of insulin immunolabelled area to the whole pancreas at the 18.5^th^ embryonic day (E18.5) (Figure S1I). In consistent, *Npc1*^-/-^ mice demonstrated decreased serum insulin level compared to WT and* Npc1*^+/-^ mice (Figure 1F)*,* whereas the random blood glucose levels were comparable between the three genotypes (Figure 1G) at this age. Generally, *Npc1*^+/-^ mice shared similar phenotypes with those of WT mice.

### *Npc1* ablation induced β cell apoptosis and dysfunction with autophagosome/lysosome accumulation

**Figure 2:**
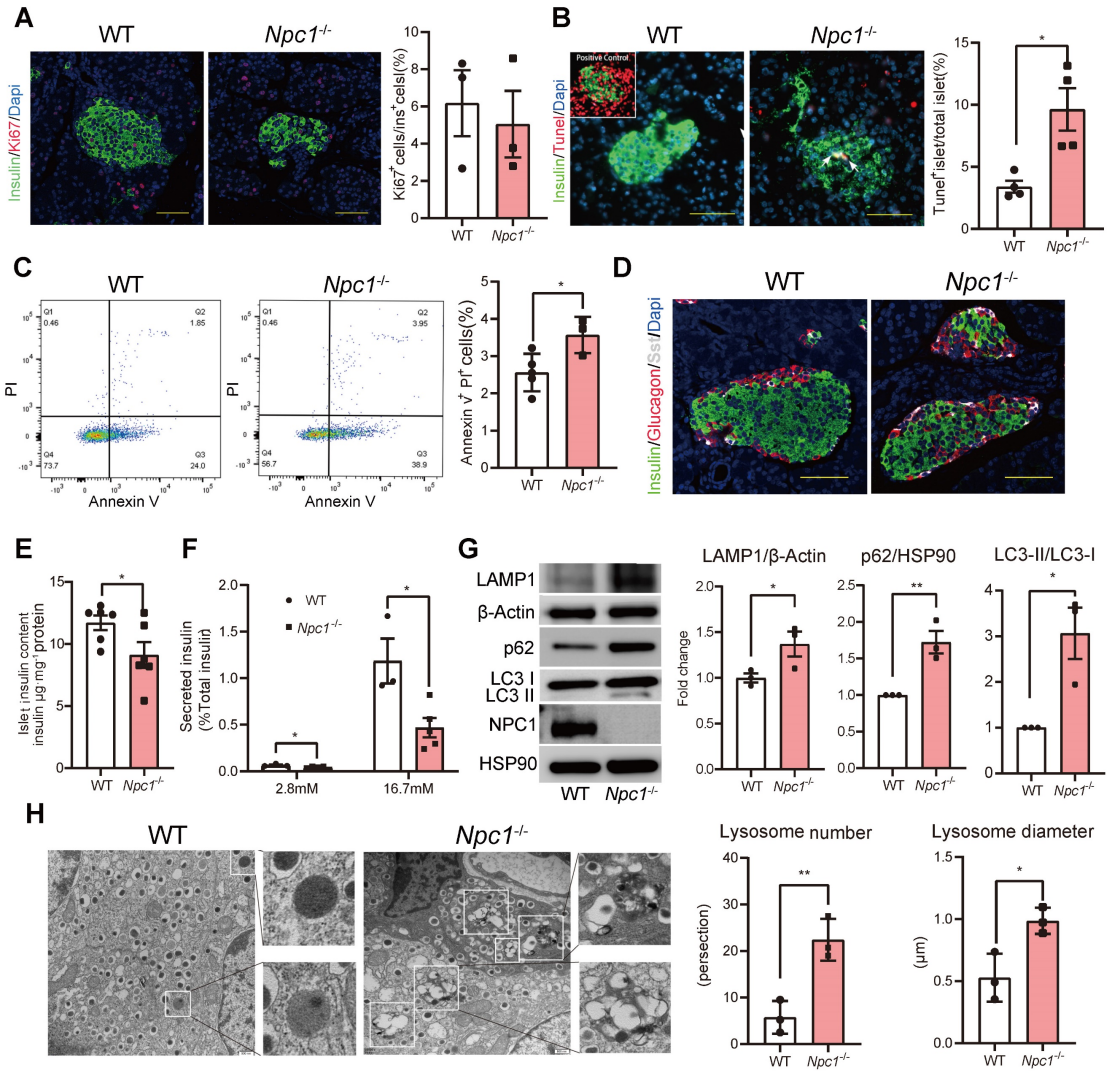
***Npc1* ablation blocks autophagy and increases neonatal β cell apoptosis. A**. β cell proliferation, left: representative images of Ki67 (red) and insulin (green) immunostaining in pancreas section. Scale bars, 50 μm; right: Percentage of Ki67^+^β cell number (count at least 2000 cells per mice, n = 3). **B**. β cell apoptosis; left: representative images of TUNEL assay for labeling apoptosis β cell in the islets from 2-week-old WT and *Npc1*^-/-^ mice; right: percent of TUNEL^+^ islets (count at least 100 islets per mice, n = 4). **C**. Flow cytometry analysis of Annexin V and PI staining of dispersed islets cells of 2-week-old WT and *Npc1*^-/-^ (n = 3-4). **D**. Islet structure, immunostaining pancreas section with glucagon (red), somatostatin (white), and insulin (green). **E**. Islet insulin content in 2-week-old WT and *Npc1*^-/-^mice (n = 6).** F**. *Ex vivo* glucose stimulated insulin secretion of primary islets from 2-week-old WT and *Npc1*^-/-^ mice treated with 2.8 and 16.7 mM glucose (normalized to total insulin content, n = 3-5). **G**. Western blot of LAMP1, LC3, p62, and NPC1 in primary islets from 2-week-old WT and *Npc1*^-/-^. **H**. Representative transmission electron microscopy images and quantification of β cells autophagosome/lysosome ultrastructure in 2-week-old WT and *Npc1*^-/-^ mice (n = 3); autophagosome/lysosomes were magnified in boxes (n = 3). Scale bars, 500 nm. Data are presented as the mean ± S.E.M, * *P* < 0.05, ** *P* < 0.01. Unpaired two-tailed Student's t test.

We sought to determine what cell biology changes knocking-out *Npc1* could induce in β cell leading to mass loss. β cell proliferation in* Npc1*^-/-^ mice, manifested by computing Ki67^+^ β cell percentage, showed no significant difference with the WT mice (Figure 2A). However, the percentage of apoptotic β cells increased in *Npc1*^-/-^ compared to WT mice, manifested by three folds increase of TUNEL labelled positive islets in* Npc1*^-/-^ mice (Figure 2B), as well as higher Annexin V/PI labeling of dispersed primary islet cells (Figure 2C). Pancreatic islet structure was moderately altered, with more centrally located non-β cells, like α and δ cells (Figure 2D). We tested if *Npc1* deletion affected β cell function and found that *Npc1*^-/-^ islets had decreased islet insulin content (Figure 2E), basal insulin secretion and nearly 70% less glucose induced insulin secretion (GSIS) in response to 16.7 mM glucose stimulation compared to WT islets after adjusted islet insulin content (Figure 2F). Hence, both decreased postnatal β cell mass growth and insufficient GSIS in *Npc1*^-/-^ mice suggested deficient β cell postnatal maturation and development.

The functional and survival defects in β cells can be induced by the blocked autophagy flux manifested by higher ratio of LC3-II to LC3-I, LAMP1 and p62 protein levels in primary *Npc1*^-/-^ islets as in other organs (Figure 2G, S1J), for loss of NPC1 not only induced a significantly elevated intracellular cholesterol content (Figure S1K), but also increased intracellular number and size of autophagosome/lysosome in β cells observed under EM (Electron microscope) with accumulating undigested autophagic substrate and intracellular cholesterol (Figure 2H). The autophagy flux in *Npc1*^-/-^ islets can be further blocked after bafilomycin A1 (BafA1) treatment, suggesting that the BafA1 treatment shared synergistical but independent effects with *Npc1* ablation in blocking autophagy (Figure S1L).

### scRNA sequencing demonstrated insufficient β cell differentiation and maturation with impaired mitochondrial respiration

**Figure 3:**
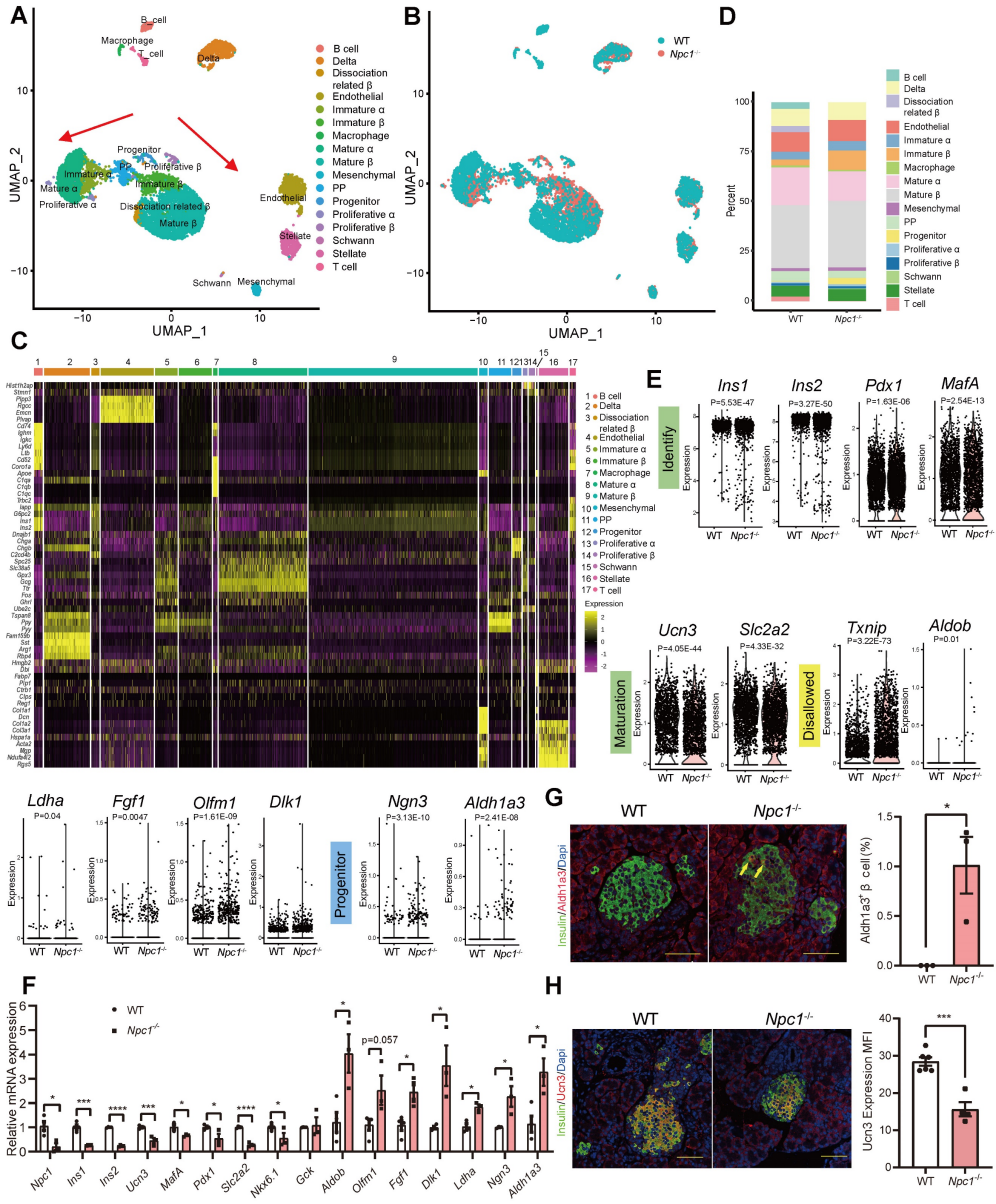
** scRNA sequencing demonstrates functional immaturity in *Npc1*^-/-^ β cells. A**. Different cell clusters and **B**. Different genotypes of Uniformed Manifold Approximation and Projection (UMAP) plot for visualizing annotated clusters in single islet cell of in 2-week-old WT and *Npc1*^-/-^ mice. Arrows depict the trajectory of transcriptional progression of endocrine cell differentiation. **C-D**. The major marker gene expression and cell number percentage of 17 different cell clusters. **E**. Violin plots for expression of identity, maturation, disallowed and progenitor genes in single 2-week-old WT and *Npc1*^-/-^ β cells. **F**. RT-PCR analysis verifies the expressions of identity, maturation, disallowed and progenitor genes in WT and *Npc1*^-/-^ islets. **G**. Representative images of Aldh1a3 (red) and insulin (green) immunostaining in pancreas section and quantification. **H**. Representative images of Ucn3 (red) and insulin (green) immunostaining in pancreas section and quantification. Scale bars, 50 μm. Data are presented as the mean ± S.E.M, * *P* < 0.05, ** *P* < 0.01, *** *P* < 0.001, **** *P* < 0.0001. Unpaired two-tailed Student's t test.

Next, we explored the potential mechanistic linkage that could mediate the effect of disturbed autophagy in normal β cell functional maturation and mass growth. Single cell RNA sequencing analysis was conducted on dispersed primary *Npc1*^-/-^ and WT islet cells in 2-week-old mice. After filtering and processing, we used data from 9,003 cells for cell clustering analysis, the islet cells were clustered into the following 17 groups (Figure 3A). *Npc1* knockout demonstrated different distribution with decreased portion of mature β and α cells but increased portion of progenitors compared to the WT islet cells (Figure 3B). Increased expression of those markers for PP cells defines β and α cells as their immature versions (Figure 3C), including *Tspan8*, *Ppy* and *Pyy*. Cell count showed that proportions of progenitor cells were increased 8 folds and immature β cells increased 3 folds in *Npc1*^-/-^ islets (Figure 3D). Additionally, in whole β cell clusters, we also found a significant decrease in the mRNA expression of β cell identity and function related genes (*Ins1*, *Ins2*, *Pdx1*, *Ucn3*, *MafA*, *Slc2a2*, *Nkx6.1*) in *Npc1*^-/-^ islet β cell. Meanwhile, we found significantly increased expression of *Txnip* and disallowed genes (*Aldob*, *Ldha*, *Fgf1*, *Dlk1*, *Olfm1*) in *Npc1*^-/-^ islets that indicated increased cell apoptosis and retarded β cell differentiation 28, 29. This might explain the previous observations of changes in β cell function and apoptosis in *Npc1*^-/-^ mice (Figure 3E). Furthermore, pancreatic progenitor markers, such as *Ngn3* and *Aldh1a3* were also significantly increased in β cells. We verified these changes in mRNA from primary islets (Figure 3F). Protein level of ALDH1A3, the major β cell dedifferentiation marker was elevated in islet lysates and ALDH1A3 positive β cells were significantly enriched in pancreas section of *Npc1*^-/-^ mice (Figure 3G, S2A). The β cells maturation marker of Urocortin3 (UCN3) was otherwise significantly attenuated in *Npc1*^-/-^ islet β cells (Figure 3H). Hence, scRNA seq analysis not only suggested the dysfunction and increased apoptosis phenotypes of *Npc1*^-/-^ pancreas islets, but further indicated a less differentiated and immature status of neonatal *Npc1*^-/-^ β cells.

**Figure 4:**
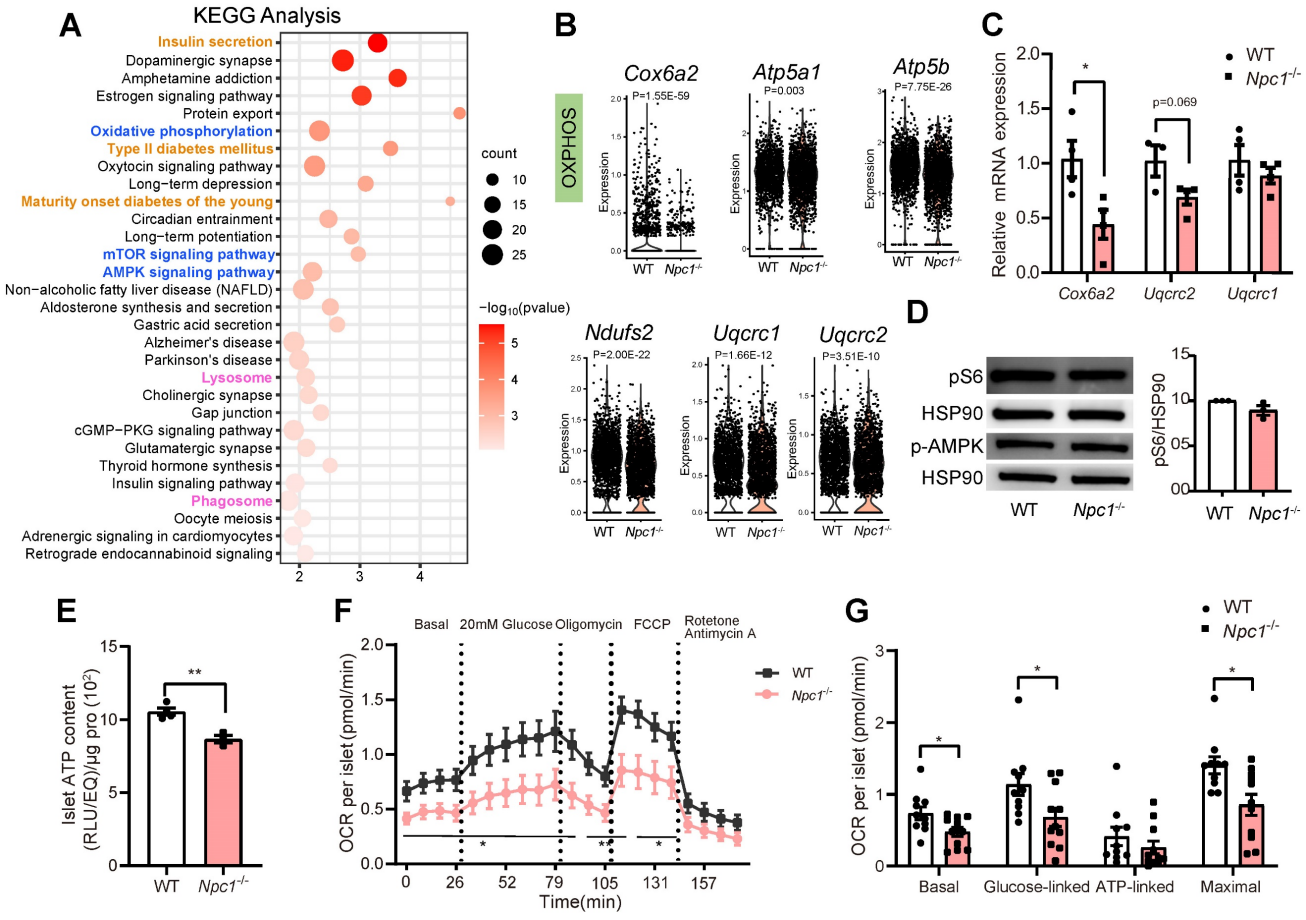
** Mitochondrial dysfunction occurs in *Npc1*^-/-^ β cells. A**. Kyoto Encyclopedia of Genes and Genomes (KEGG) analysis of differentially expressed genes (DEGs, *P*_adj_ < 0.01) between single WT and *Npc1*^-/-^ β cells.** B**. Violin plot for expression of oxidative phosphorylation (OXPHOS) genes in single WT and *Npc1*^-/-^ β cells. **C**. RT-PCR analysis of OXPHOS gene in WT and *Npc1*^-/-^ primary islets (n = 3-4). **D**. Western blot and quantification for pS6 and p-AMPK levels in WT and *Npc1*^-/-^ primary islets. **E**. ATP content of primary islets from 2-week-old WT and *Npc1*^-/-^ mice (n = 3-4). **F-G**. Oxygen consumption rate (OCR) analysis of WT and *Npc1*^-/-^ islets (n = 10-11). Data are presented as the mean ± S.E.M, * *P* < 0.05, ** *P* < 0.01, unpaired two-tailed Student's t test.

KEGG pathway analysis of DEGs in β cell population showed that in addition to altered expressions of genes regulating phagosome/lysosome, insulin secretion and T2D, pathways regulating mTOR/AMPK signaling and oxidative phosphorylation were also affected (Figure 4A). RT-PCR verification of whole islet lysates showed the similar trends of expressional changes of genes regulating the respiration chain and oxidative phosphorylation, such as *Cox6a2, Uqcrc1, Uqcrc2, Atp5a1,* and *Atp5b* in *Npc1*^-/-^*vs* WT (Figure 4B-C), whereas the protein levels of mitochondrial respiration complex (Figure S2B) or pS6/p-AMPK levels were not significantly altered (Figure 4D). Despite the inconsistence of translational and transcriptional changes, total islet ATP content (Figure 4E) as well as mitochondrial oxidation function in primary* Npc1*^-/-^ islets measured by Seahorse XF24 were significantly reduced compared to the control littermates both in basal and glucose induced OCR (Figure 4F-G).

### *Npc1*^-/-^ Min6 cells exhibited similar changes with primary *Npc1*^-/-^ β cells

**Figure 5:**
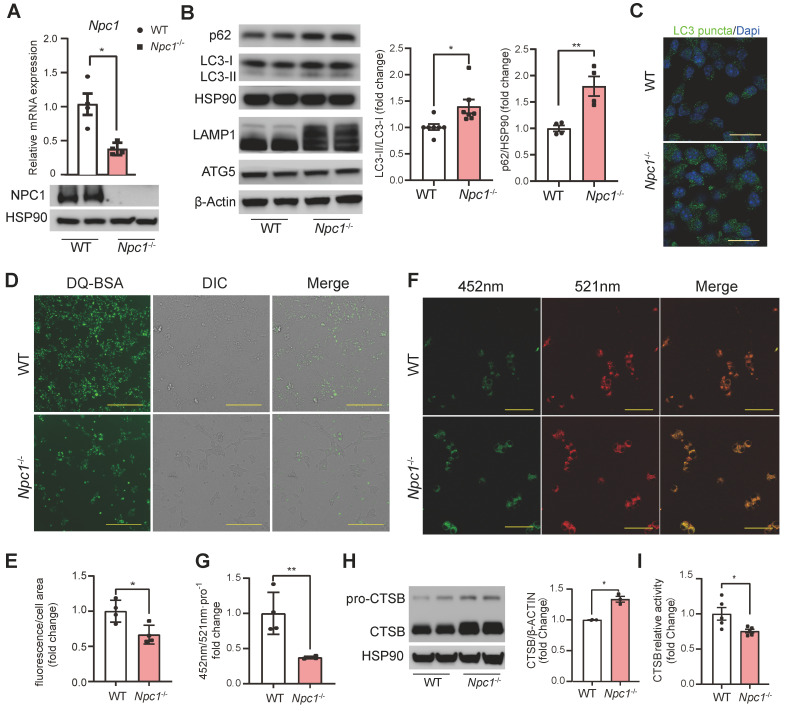
***Npc1* knockout Min6 cell had decreased proteolytic function. A**. RT-PCR and Western blot of *Npc1* expression in WT and *Npc1*^-/-^ Min6 cells (n = 4). **B**. Western blot and analysis of autophagy flux: LC3, p62, LAMP1 and autophagy initiating proteins: ATG5 expression in WT and *Npc1*^-/-^ Min6 cells. **C**. Representative images of LC3 immunostaining in WT and *Npc1*^-/-^ Min6 cells. Scale bars, 20 μm. **D-E.** Representative images and fluorescence quantitation of DQ-BSA; fluorescent intensity increases as it is transported to the lysosome and digested (n = 4). Scale bars, 200 μm. **F-G**. Representative images and fluorescence quantitation of lysosome pH value, which was calculated by dividing fluorescence intensity at excitation 335nm emission 452 nm (green, G) and excitation 381nm emission 521 nm (red, R), when lysosome pH decreases, R/G ration would increase (n = 4). Scale bars, 50 μm. **H**. Western blot of CTSB expression in WT and *Npc1*^-/-^ Min6 cells. **I**. Relative proteolysis enzymatic activity of CTSB in WT and *Npc1*^-/-^ Min6 cells (n = 5). Data are presented as the mean ± S.E.M, * *P* < 0.05, ** *P* < 0.01, *** *P* < 0.001. Unpaired two-tailed Student's t test.

To further verify and study the altered intracellular signaling found by scRNA seq in *Npc1* null β cells, we used CRISPR/Cas9 editing to generate *Npc1* knockout (*Npc1*^-/-^) Min6 cells. Western blot and RT-PCR confirmed deletion of *Npc1* (Figure 5A). Similar in *Npc1*^-/-^ islets, *Npc1*^-/-^ Min6 cells showed increased p62, LC3II/I, LAMP1 levels and LC3 puncta, with unaffected protein levels of ATG5, demonstrating that* Npc1* deletion increased autophagosome/lysosomes and blocked autophagy flux in Min6 cells (Figure 5B-C). The blocked autophagy flux was exacerbated by BafA1 treatment to a larger extend in *Npc1*^-/-^ than WT Min6 cells (Fig S3A). *Npc1*^-/-^ Min6 cells also exhibited lower intracellular DQ-BSA uptake (Figure 5D-E), increased lysosome pH in *Npc1*^-/-^ Min6 cells (Figure 5F-G) and decreased Cathepsin B (CTSB) activity albeit with increased protein level (Figure 5H-I), all these results indicated lowered lysosome digest function 30. Thus, despite having increased lysosome quantity, the digestion function of lysosome in *Npc1*^-/-^ cells was compromised, which hence blocked the autophagy flux in β cells.

**Figure 6:**
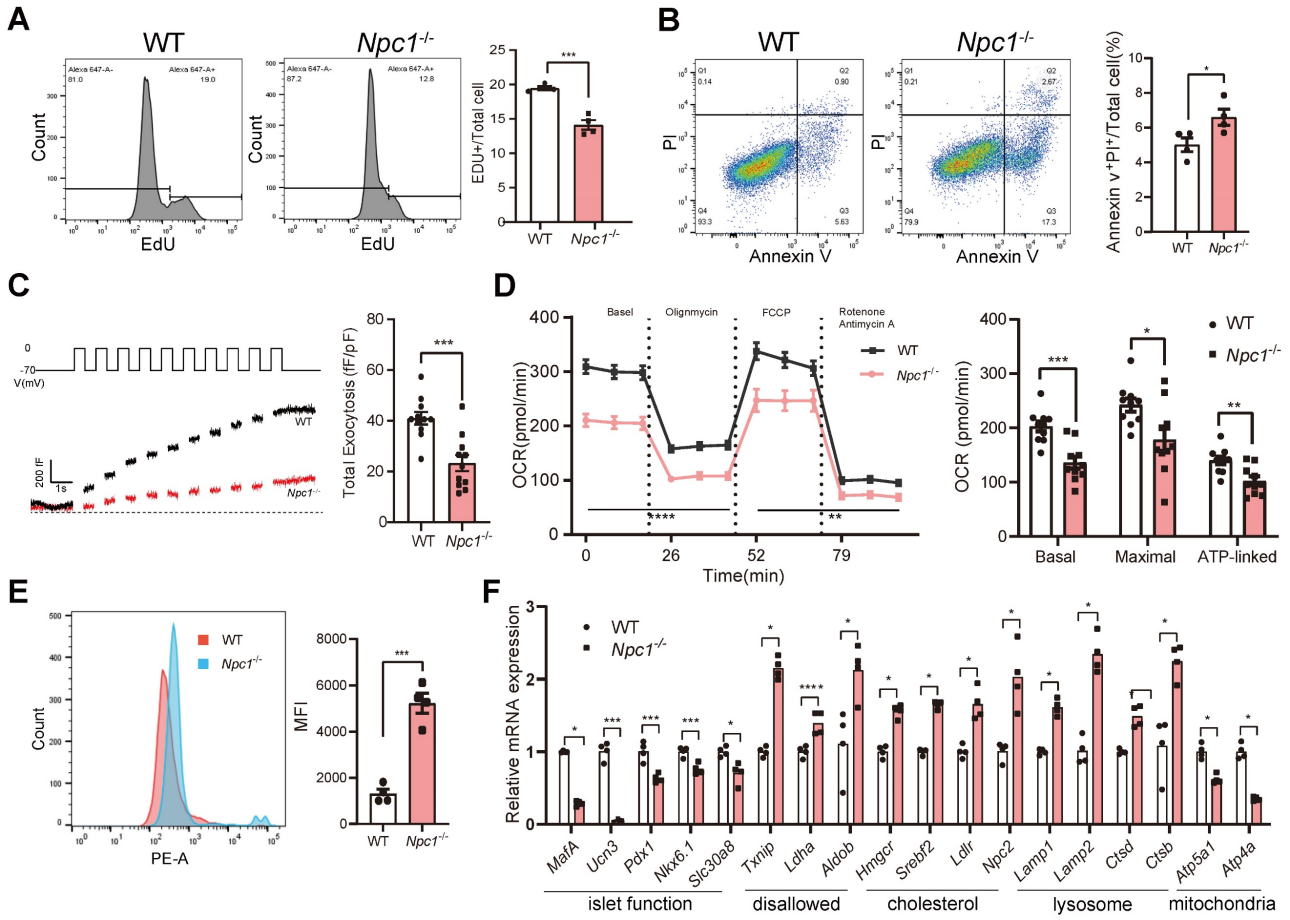
*** Npc1* knockout Min6 cell shared similar changes with primary *Npc1^-/-^* islet β cells. A**. Flow cytometry analysis of EDU labelled WT and *Npc1*^-/-^ Min6 cells (n = 4). **B**. Flow cytometry analysis of Annexin V and PI labeled WT and *Npc1*^-/-^ Min6 cells (n = 4).** C**. Exocytosis (measured as cumulative increase in membrane capacitance, ΔCm) elicited by a train of ten 500ms depolarizations from -70 to 0 mV applied to WT and *Npc1*^-/-^ Min6 cells to 20mM glucose **I**. Quantification of ∑ΔCm from WT and *Npc1*^-/-^ Min6 cells following acute 20mM glucose treatment (n = 11). **D**. OCR analysis of WT and *Npc1*^-/-^ Min6 cells (n = 12). **E**. Flow cytometry analysis of MitoSOX level in WT and *Npc1*^-/-^ Min6 cells (n = 4). **F**. RT-PCR analysis of identity, maturation, disallowed, cholesterol, lysosome, and OXPHOS gene of WT and *Npc1*^-/-^ Min6 cells (n = 3-4). Data are presented as the mean ± S.E.M, * *P* < 0.05, ** *P* < 0.01, *** *P* < 0.001. Unpaired two-tailed Student's t test.

Except for decreased cell proliferation (Figure 6A), similarly with primary islet β cells, Min6 cells lacking *Npc1* exhibited increased cell apoptosis (Figure 6B), attenuated glucose induced insulin exocytosis measured by glucose induced membrane capacitance changes (Figure 6C), and reduced glucose induced OCR in *Npc1*^-/-^ Min6 cells (Figure 6D). Additionally, mitochondrial superoxide productions were significantly increased in *Npc1*^-/-^ Min6 cells (Figure 6E). Aligned with the above results and the scRNA seq analysis of primary β cells, higher expression of disallowed genes (mostly glycolytic genes), genes regulating cholesterol synthesis and uptake as well as lysosome genes, including *Lamp1, Lamp2, Ctsb,* and those encoding mitochondria respiration chain were all significantly changed in *Npc1*^-/-^ Min6 cells (Figure 6F). However, like in primary islets, no changes in respiration chain protein levels were found (Figure S3B), nor the changes of mitochondria quantity (COX4 and mtDNA) (Figure S3C-D). Therefore,* Npc1*^-/-^ Min6 cells largely phenocopied the primary *Npc1*^-/-^ β cells and exhibited reduced lysosome digest function and disrupted mitochondria OXPHOS function, without affecting OXPHOS proteins level or mitochondrial quantity.

### *Npc1* knockout disturbed mitophagy and promoted apoptosis in β cells

**Figure 7:**
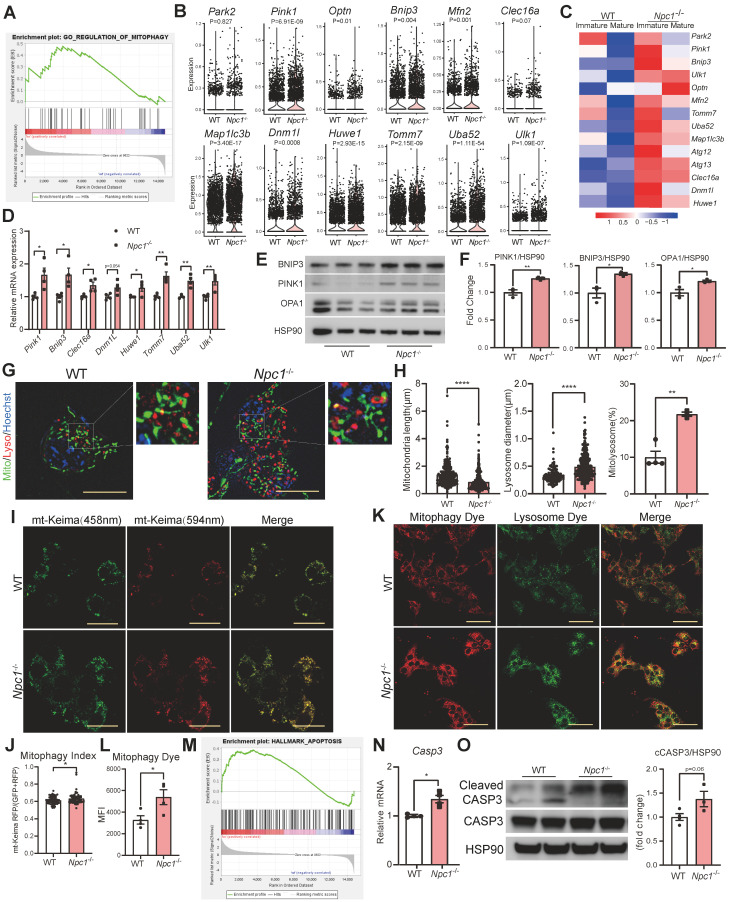
***Npc1* knockout disturbed normal mitophagy in β cells. A**. GSEA of genes regulating mitophagy in WT and *Npc1*^-/-^ islet β cell scRNA-seq data. **B**. Violin plots of mitophagy-related gene expression in WT and *Npc1*^-/-^ β cells. **C**. Heatmap of the average mitophagy gene expression in scRNA seq data from mature and immature WT and *Npc1*^-/-^ β cells. **D**. RT-PCR analysis of Mitophagy gene of WT and *Npc1*^-/-^ Min6 cells (n=3-4). E-F. Western blot and quantitation of PINK1, BNIP3 and OPA1 in WT and *Npc1*^-/-^ Min6 cells. **G**. Representative ultrahigh resolution microscopy images of lysosome(red), mitochondrion (green) and nucleus (blue) in WT and *Npc1*^-/-^ Min6 cells. Scale bars, 10 μm. **H**. Quantification of mitochondria length (> 200 mitochondria), lysosome diameter (> 200 lysosomes) and percentage of mitolysosome/lysosome (mitochondria colocalized in lysosome, per section, > 10 cells per group) in WT (4 images with 2-6 cells per image) and *Npc1*^-/-^ Min6 cells (3 images with 7-8 cells per image). **I**. Representative images of mt-Keima fluorescence 594 nm(red) and 458 nm (green) and J. Mitophagy index quantification in WT and *Npc1*^-/-^ Min6 cells. Scale bars, 20 μm. **K.** Representative images, and **L.** Quantitation of Mitophagy assay fluorescence staining (Mitophagy Dye, fluorescence intensity of which increases as mitochondria undergo mitophagy process) in WT and *Npc1*^-/-^ Min6 cells. Scale bars, 50 μm. **M.** GSEA analysis of apoptosis pathway in WT and *Npc1*^-/-^ islet β cell scRNA-seq data. **N.** RT-PCR analysis of *Casp3* gene of WT and *Npc1*^-/-^ Min6 cells (n = 4). **O.** Western blot and quantification for the expression of Cleaved Casp3 and Casp3 in WT and *Npc1*^-/-^ Min6 cells. Data are presented as the mean ± S.E.M, * *P* < 0.05, ** *P* < 0.01, **** *P* < 0.0001. unpaired two-tailed Student's t test.

Based on the above changes, the disrupted mitochondria respiration could be resulted by disrupted selective autophagy of mitochondria: mitophagy 32, 33. GSEA of scRNA sequencing data showed that the mitophagy pathway was significantly activated in *Npc1*^-/-^ β cells (Figure 7A). The transcriptional expression levels of genes such as *Pink1*,* Bnip3 and Optn,* etc were elevated in *Npc1*^-/-^ β cells and immature WT β cells (Figure 7B-C), which were also found declined along with β cell maturation in another published single cell sequencing data (Figure S4A) 31, implying the mitophagy activity was related with the β cell maturation. We then confirmed the transcriptional and protein level changes of mitophagy proteins in *Npc1*^-/-^ Min6 cells (Figure 7D-F). CCCP treatment elevated the expression of mitophagy proteins in WT Min6 cells and this response was attenuated in *Npc1*^-/-^ Min6 cells (Figure S5A-B).

With the aid of ultrahigh resolution Lattice SIM^2^ (Figure 7G), we found that with the significantly increased size, number of lysosomes, and shortened mitochondria, the colocalization of mitochondria with lysosome in *Npc1*^-/-^ Min6 cells was significantly enhanced (Figure 7H). This lysosome colocalized mitochondria has recently been recognized as “Mitolysosome” 32. Both mt-Keima test and mitophagy dye assay 22, 33, 34 confirmed elevated mitophagy flux in *Npc1*^-/-^ Min6 cells (Figure 7I-L). However, the percentage of mitochondria localized LC3 puncta were not significantly altered in *Npc1*^-/-^Min6 cells, despite of increased total LC3 puncta counts (Figure S5C-D). These changes further suggested that the mitophagy in *Npc1*^-/-^ β cells was blocked in the stage of lysosome degradation 35 and the elevated mitophagy proteins induced by *Npc1* deletion was rather a feedback response and unable to futher increase mitophagy flux.

Dysfunction mitochondria OXPHOS or increased mitochondria superoxide could intervene both cell differentiation and survival. GSEA analysis also showed that intrinsic apoptosis pathway was significantly enhanced in *Npc1*^-/-^ islet β cells (Figure 7M) and Cleaved Caspase 3 in protein lysates of *Npc1*^-/-^ Min6 cells was elevated (Figure 7N-O). Thus, we confirmed that loss of NPC1 in β cell blocked mitophagy that mainly impaired mitochondrial respiration and promoted mitochondria derived cell apoptosis. To further identify the potential signal linking NPC1 loss indued mitochondria incompetence to cell fate or survival changes, we tested the integrated stress response (ISR) activity 36-38. However, we found no overall specific transcriptional alteration patterns of ISR genes in scRNA sequencing between mature and immature β-cell, or between WT and *Npc1*^-/-^ Min6 cells, and p-eIF2α level, the main manifestation of ISR activity was unaltered between WT and *Npc1*^-/-^ Min6 cells (Figure S6).

## Discussion

**Figure 8:**
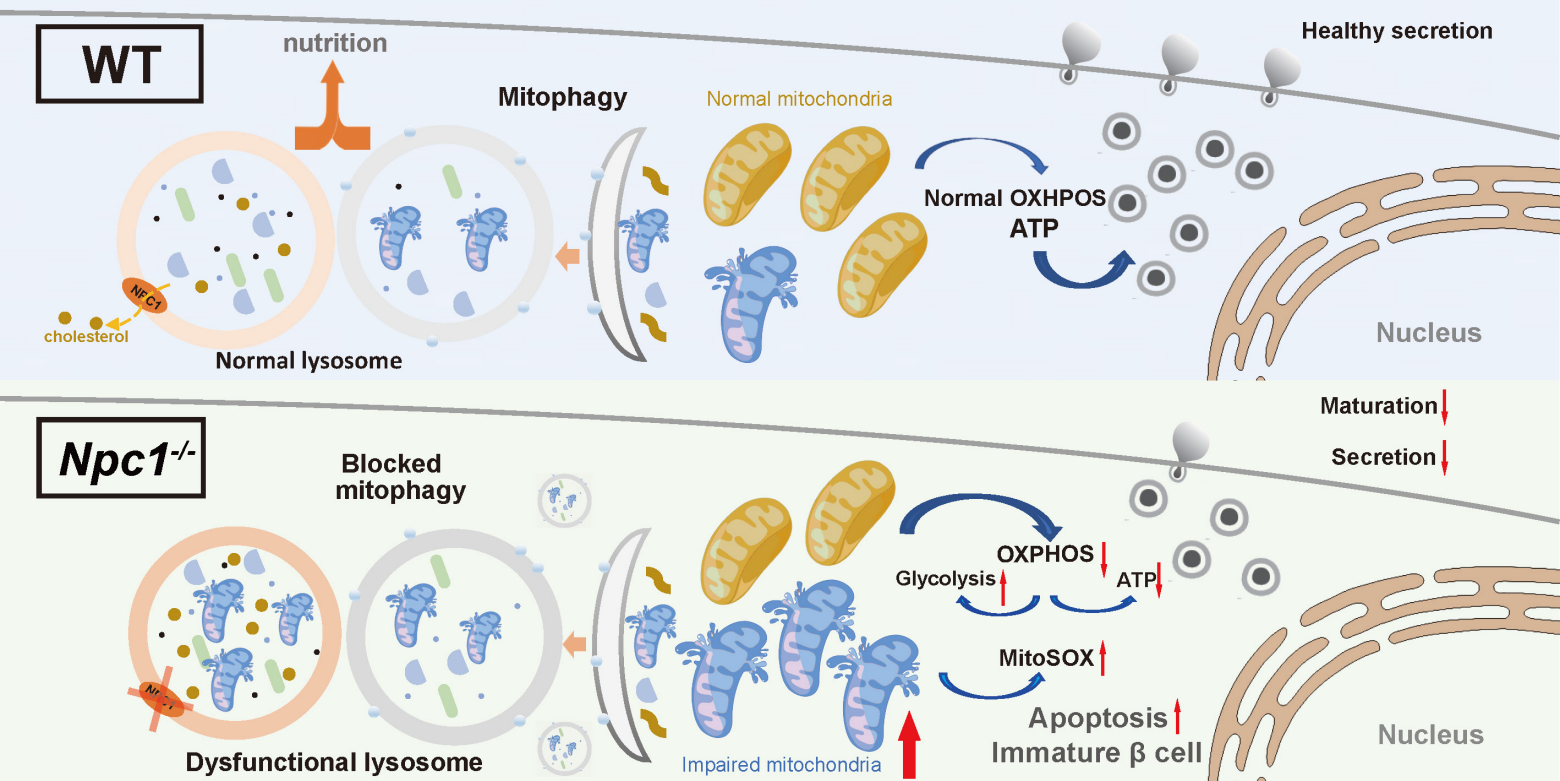
**Illustration of the mechanism underlying the role of NPC1 in β cell maturation.** NPC1 locates on the lysosomal membrane of islet β cells, transports cholesterol and maintains the normal function of lysosomes. The deletion of NPC1 leads to the accumulation of cholesterol in the lysosome, which decreases lysosomal hydrolysis function. The normal process of mitophagy is whereby affected and dysfunctional mitochondria are accumulated in β cells. Finally, the normal mitochondrial function is undermined and normal β cell maturation is deferred. Created with Figdraw.com.

In this study, we for the first time revealed the effect of NPC1 on the postnatal pancreas β cell differentiation. In 2-week-old *Npc1* global knockout mice, we observed reduced β cell mass and GSIS with increased cell apoptosis. Sc-RNA seq data demonstrated expression of genes regulating β cell maturation, lysosome function, and mitochondrial respiration were altered. In *Npc1*^-/-^ Min6 cells, we verified that *Npc1* depletion blocked mitophagy by impairing lysosome function to disrupt mitochondrial respiration, pancreatic β cell maturation and survival (Figure 8).

NPC1 loss induces LSDs with the accumulation of undegraded autophagosome/lysosomes, which decreases lysosomal proteolytic function. In recent years, lysosomes have been found to play active role in multiple cellular processes other than degrading cellular wastes, such as signal transduction, material processing, energy sensing, cell membrane repairing, cytokine secretion and autophagy flux 8. Autophagy maintains the healthy circulation of organelles and other cell components as well as cell nutrient sensing to maintain metabolic homeostasis and normal function. Abnormal autophagy induces ER stress, the unfolded protein response and precluded organelle degradation, such as mitophagy, ERphagy, pexophagy as well as amyloid protein accumulation 16, 39. Both inhibiting or enhancing autophagy in β cells by knocking out *Atg7* (low autophagy) or overexpressing *Becn1* (high autophagy) impair β cell function and lead to a diabetic phenotype 40, 41. Here, we first demonstrated the correlation of lysosome dysfunction and blocked autophagy flux with disturbed mitophagy that could exert detrimental effect in postnatal β cell differentiation and maturation. Autophagy of other organelles could be involved, which requires future study to confirm.

Mitophagy is important for mitochondria turnover and healthy mitochondrial function 42. Blocked mitophagy flux confers accumulated dysfunctional mitochondria 43 that bear decreased oxidative phosphorylation, less ATP synthesis and increased superoxide production, which not only compromises matured insulin secretion function, but also provokes apoptosis and impairs β cell maturation 44-46. After in depth molecular and cell biology studies, we were able to confirm that the mitophagy flux was blocked in the lysosome digestion stage in *Npc1*^-/-^ β cells, and delineated that the elevated expressions of mitophagy activation machinery proteins could work as the feedback response against the blocked mitophagy flux and be insensitive to additional stimulation of mitophagy by CCCP 35. We thought the mitochondria dysfunction secondary to blocked mitophagy resulted in elevated cell apoptosis and delayed postnatal β cell differentiation during β cell weaning.

Considering its requirement for adult stem cells differentiation of other tissues 47, 48, we think the normal mitophagy flux could be the main downstream target of NPC1 relating lysosome function regulation to serve as a key trigger to postnatal β cell maturation in addition to maintaining normal β cell survival and nutrient coupled insulin secretion 49-53. To support this notion, scRNA seq data in *Npc1*^-/-^ β cell demonstrated an increased proportion of immature β and progenitor cell populations, decreased key pancreatic transcription factors and increased expression of disallowed genes (majorly enhancing glycolysis) in *Npc1*^-/-^ β cells, indicating that due to disturbed mitophagy, accumulated unhealthy mitochondria was unable to carry on sufficient glucose oxidation and to promote cell metabolic reprogramming that underlines postnatal β cell differentiation. Mitochondria respiration linked integrated stress response (ISR) plays a key role in embryogenesis and cell development in lung and brain 36-38, hence we guessed that ISR could mediate the mitochondrial dysregulation induced by loss of NPC1 to nucleus to drive reprogram. However, we did not find altered p-eIF2α levels and significant altered expression pattern of ISR genes in scRNA seq between WT versus *Npc1*^-/-^ or mature versus immature β cells. On the other hand, lysosome leakage induced by the accumulated autophagsome/lysosome can prompt cell apoptosis 54, and lysosome relating signals have been known pivotal for cell fate decision 55-57. Therefore, future studies should be conducted to investigate other signals mediating NPC1 related mitophagy, from both mitochondria and lysosome to the expression of key pancreatic transcription genes, such as *Pdx1*, *MafA, Nkx6.1,* etc*.*

Activity switch of essential intracellular nutrient sensors AMPK/mTORC1 has been suggested to regulate metabolic reprogram 20, 58, 59 underlining the postnatal β cell development. Both signaling pathways require lysosomal localization to exert their functions 15, 60, 61. Conversely, AMPK/mTORC1 activities are important for the biosynthesis and activity of both mitochondria and lysosomes for initiating autophagy 62, 63. Recent studies have found that mTORC1 activity can be controlled by NPC1 to sense the cholesterol levels in lysosome 64, implying the potential of lysosome/mTORC1 mediated cholesterol regulation on the β cell maturation. However, we did not find altered mTORC1 and AMPK activities in *Npc1*^-/-^ and WT Min6 cells, suggesting that NPC1/lysosome regulation might work downstream of these two nutrient sensors in neonatal β cells.

Despite impacting mitochondrial function, lysosome mediated autophagy also regulates different forms of vesicle degradation under diverse nutritional statuses, such as excretion lysosome, crinophagy, SINGD (starvation-induced nascent degradation), etc. in pancreatic islet cells 65, 66. Most of these studies suggest that lysosome plays a surveillance role in restraining unnecessary insulin secretion under fasting condition. However, in these* Npc1*^-/-^ β cells we found a deficient GSIS rather than increased fasting insulin secretion, which was most possibly dependent on insufficient β cell maturation and insulin biosynthesis.

Our study had below limitations. We did not include the investigation on β cell specific Npc1 knockout mice. An inducible organ specific model could be helpful to delineate how NPC1 is required for the physiology of β cell in different development stages. We did not evaluate the homeostasis of other organelles, such as ER, peroxisome, and Golgi which could also be affected by loss of NPC1 induced autophagy flux blockage.

In this study, we demonstrated that NPC1 in β cells could maintain normal lysosome function, one of major subsequent intracellular regulation of which was to sustain mitophagy flux to permit mitochondria OXPHOS mediating the metabolism reprogram that is essential for neonatal β cell maturation and survival. This study explored a new angle for investigating the role of mitochondria dynamics in postnatal pancreatic islet development. Elucidating the role of NPC1 and related lysosome function in β cell differentiation regulation will facilitate the discovery of new drug targets for T2D as well as Nieman Pick disease.

## Supplementary Material

Supplementary figures and table.

## Abbreviations

NPC1: Niemann-Pick disease type C intracellular cholesterol transporter 1
T2D: type 2 diabetes
eQTL: expression quantitative trait locus
CRISPR: clustered regularly interspaced short palindromic repeats
OXPHOS: oxidative phosphorylation
MitoSOX: mitochondrial superoxide
LSD: lysosomal storage disorder
SNP: single nucleotide polymorphism
GWAS: genome wide association study
HFD: high fat diet
AMPK: adenosine 5'-monophosphate (AMP)-activated protein kinase
mTOR: mechanistic target of rapamycin
mTORC1: mTOR Complex1
BafA1: bafilomycin A1
CCCP: carbonyl cyanide-m-chlorophenylhydrazone
LAMP1: lysosomal associated membrane protein 1
ATG5: autophagy related protein 5
CTSB: cathepsin B
LC3: microtubule-associated protein 1 light chain 3
p62: sequestosome 1, SQSTM1
pS6: phosphorylation protein S6
COX4: cytochrome C oxidase subunit IV
CHOP: C/EBP-homologous protein
OPA1: optic atrophy 1
PINK1: PTEN induced putative kinase 1
ALDH1A3: aldehyde dehydrogenase family 1, subfamily A3
p-eIF2α: phosphorylation eukaryotic translation initiation factor 2A
eIF2α: eukaryotic translation initiation factor 2A
HSP90: heat shock protein 90
UCN3: urocortin3
GCG: Glucagon
SST: Somatostatin
DQ-BSA: dye quenched-bovine serum albumin
MtDNA: mitochondrial deoxyribonucleic acid
CОX2: cytochrome oxidase subunit 2
OCR: oxygen consumption rate
FCCP: fluoromethoxy carbonyl cyanide phenylhydrazone
EPC: electrophysiological patch clamp
Ngn3: neurogenin3
DEGs: differentially expressed genes
KEGG: Kyoto Encyclopedia of Genes and Genomes
TUNEL: TdT-mediated dUTP nick end labeling
PI: propidium iodide
GSIS: glucose-stimulated insulin secretion
EM: electron microscope
GSEA: gene set enrichment analysis
scRNA seq: single cell RNA sequencing
PP cell: pancreatic polypeptide cell
Tspan8: tetraspanin 8
Ppy: pancreatic polypeptide
Pyy: peptide YY
Pdx1: pancreatic and duodenal homeobox 1
MafA: MAF BZIP transcription factor A
Slc2a2: solute carrier family 2 member 2
Glut2: glucose transporter 2
Nkx6.1: NK6 homeobox 1
Txnip: Thioredoxin-interacting protein
Aldob: fructose bisphosphate
Ldha: lactate dehydrogenase
Fgf1: fibroblast growth factor 1
Dlk1: delta like non-canonical notch ligand 1
Olfm1: olfactomedin 1
Cox6a2: cytochrome C oxidase subunit 6A
Uqcrc1: ubiquinol-cytochrome C reductase core protein 1
Uqcrc2: ubiquinol-cytochrome C reductase core protein 2
Atp5a: ATP synthase F1 subunit alpha
Atp5b: ATP synthase F1 subunit beta
Lamp2: lysosomal associated membrane protein 2
Optn: optineurin
Atg7: autophagy related 7
Becn1: beclin 1
SINGD: starvation-induced nascent degradation
EdU: 5-ethynyl-2'-deoxyuridine
