# Supplementary material for: NPC1 is required for postnatal islet β cell differentiation by maintaining mitochondria turnover
Source: Theranostics. 2024 Feb 24;14(5):2058–74. doi: 10.7150/thno.90946 (PMC10945349; doi:10.7150/thno.90946)
Supplement: Supplementary file 1 — Supplementary figures and table. [file thnov14p2058s1.pdf]

Supplemental Material

NPC1 is required for postnatal islet β cell differentiation by maintaining mitochondria turnover

Bei Liu[1,2*], Duanyi Hua [1,2*], Linyan Shen [1*], Tingting Li [1,2], Zheying Tao [1], Chenyang Fu [1,2], Zhongzheng Tang [1,2], Jie Yang [1,2], Li Zhang [1], Aifang Nie [1,2], Yiran Jiang [1,2], Jiqiu Wang [1,2], Yang Li [3], Yanyun Gu [1,2✉], Guang Ning [1,2✉]

1. Department of Endocrine and Metabolic Diseases, Shanghai Institute of Endocrine and Metabolic Diseases, Ruijin Hospital, Shanghai Jiao Tong University School of Medicine, Shanghai, China.
2. Shanghai National Clinical Research Center for Metabolic Diseases, Key Laboratory for Endocrine and Metabolic Diseases of the National Health Commission of the PR China, Shanghai National Center for Translational Medicine, Ruijin Hospital, Shanghai Jiao Tong University School of Medicine, Shanghai, China.
3. Department of Pharmacology, State Key Laboratory of Medical Neurobiology and MOE Frontiers Center for Brain Science, Key Laboratory of Metabolism and Molecular Medicine, Ministry of Education, School of Basic Medical Science, Fudan University, Shanghai, China.

✉Correspondence: guyanyun@sjtu.edu.cn (Y. Gu); gning@sibs.ac.cn (G. Ning)

* These authors contributed equally to this work.

Address: Shanghai Institute of Endocrine and Metabolic Disease, 197 Ruijin Road II, Shanghai 200025, China.

Phone: 86.21.64370045

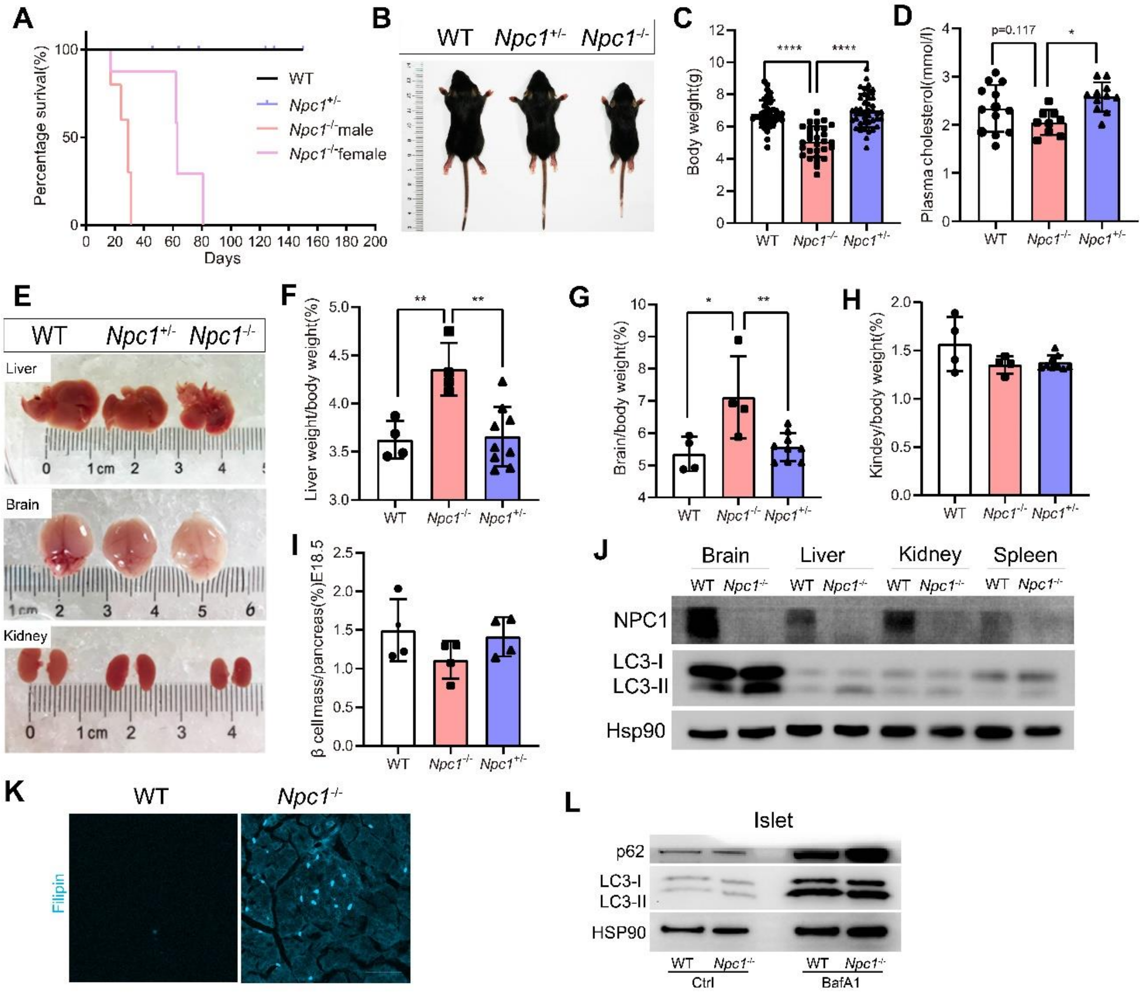

Supplemental Figure 1

A. Survival curve of studied mice (WT♂4♀4, *Npc1*$^{+/-}$ ♂4♀4, *Npc1*$^{-/-}$♂6, *Npc1*$^{-/-}$♀6) **B**. Representative images of whole 2-week-old mice **C**. Body weights of 2-week-old mice (n = 29-42) **D**. Plasma cholesterol levels in 2-week-old WT, *Npc1*$^{-/-}$, and *Npc1*$^{+/-}$ mice (n = 9-13) **E**. Representative images of the liver, brain, and kidney of 2-week-old WT, *Npc1*$^{-/-}$, and *Npc1*$^{+/-}$ mice (n = 4) **F-H**. Percentages of liver, brain, and kidney weights versus body weight of 2-week-old WT, *Npc1*$^{-/-}$, and *Npc1*$^{+/-}$ mice (n = 4) **I**. Western blot depicts the expression of NPC1, LC3-I, and LC3-II in WT and *Npc1*$^{-/-}$ mouse brain, liver, kidney, and spleen tissues **J**. Percentage of the insulin immunostaining area in the entire pancreas of E18.5 WT, *Npc1*$^{-/-}$, and *Npc1*$^{+/-}$ mice (n = 4). **K**. Representative images of Filipin stain for pancreas section in WT and *Npc1*$^{-/-}$ mice. **L**. Western blot of LC3 and p62 in WT and *Npc1*$^{-/-}$ treated with and without BafA1(20 nM, 24 h) islets. Data are presented as the mean ± S.E.M, * P < 0.05, ** P < 0.01, *** P < 0.001, **** P < 0.0001. Unpaired two-tailed Student's t test.

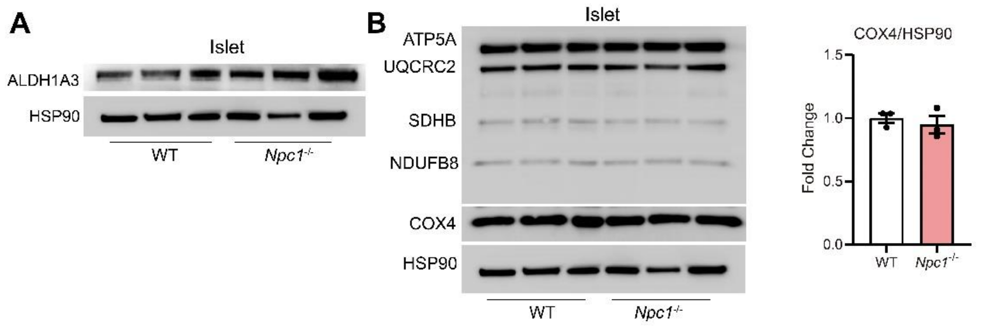

Supplemental Figure 2

A. Western blot of ALDH1A3 in WT and *Npc1*$^{-/-}$ islets. **B**. Western blot of OXPHOS protein (ATP5A, UQCRC2, SDHB and NDUFB8） and COX4 (quantification) in WT and *Npc1*$^{-/-}$ islets.

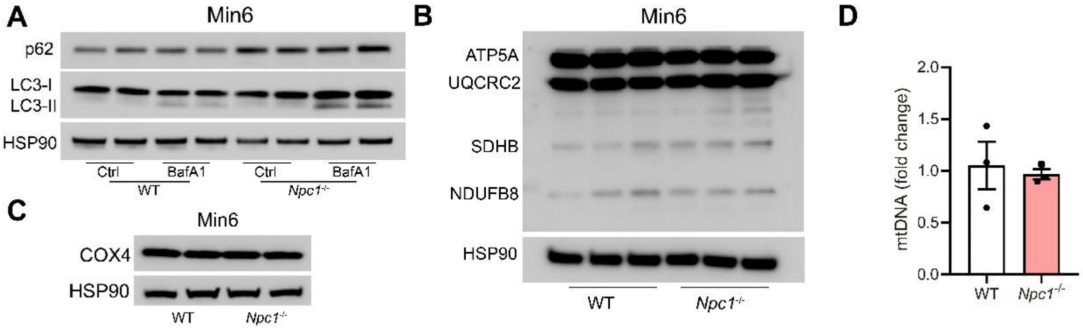

Supplemental Figure 3

A. Western blot of LC3, p62 in WT and *Npc1*$^{-/-}$ cells treated Min6 with and without BafA1 (20 nM, 24 h). **B-C**. Western blot OXPHOS protein (ATP5A, UQCRC2, SDHB and NDUFB8) in WT and *Npc1*$^{-/-}$ Min6 cells. **D**. MtDNA content in WT and *Npc1*$^{-/-}$ Min6 cells. Data are presented as the mean ± S.E.M,

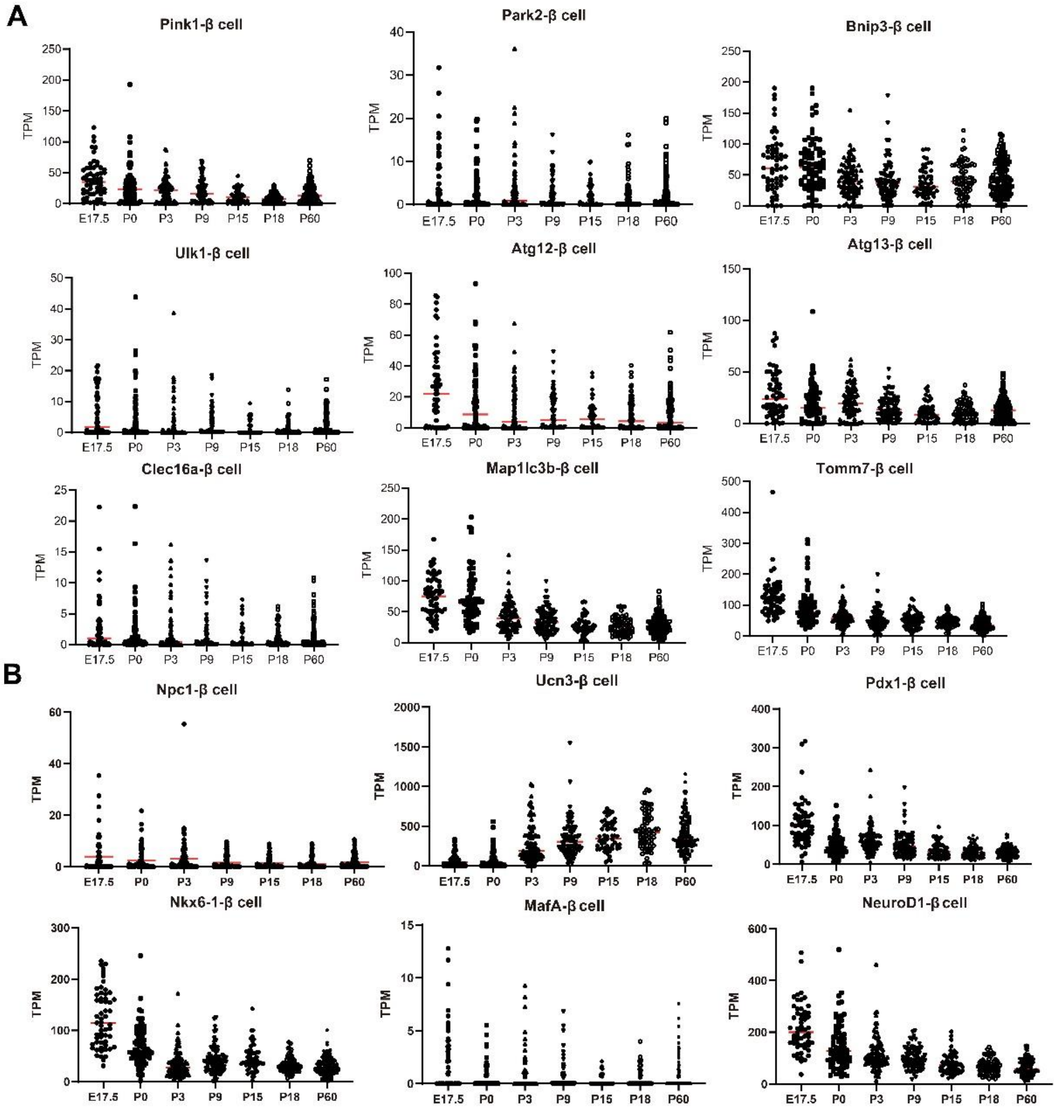

Supplemental Figure 4

A. Main mitophagy gene expression in embryonic 17.5 days to postnatal day 60. **B**. *Npc1* and islet maturation gene expression in embryonic 17.5 days to postnatal day 60. (scRNA-seq data from the GEO database GSE87375)

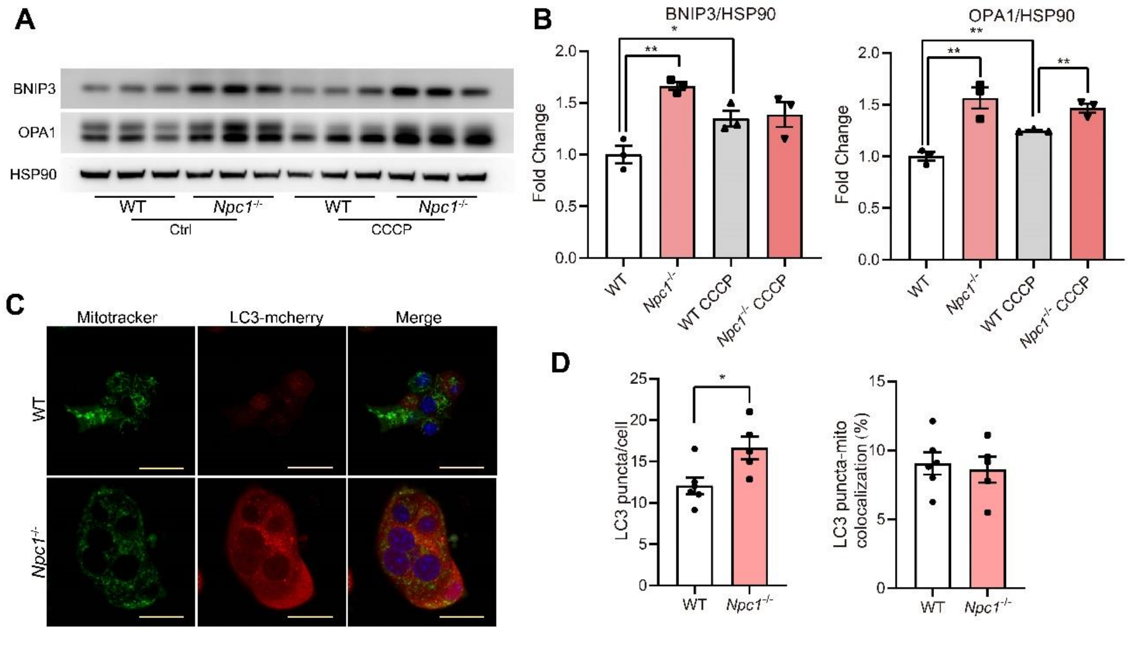

Supplemental Figure 5

A-B. Western blot and quantification of BNIP3 and OPA1 in WT and *Npc1*$^{-/-}$ treated with CCCP (1 μM, 3 h) and control vehicle on Min6 cells. **C-D**. Representative images and quantification of LC3-puncta (red) and its colocalization with Mitotracker (green) staining in WT and *Npc1*$^{-/-}$ Min6 cells. Scale bars, 20 μm. Data are presented as the mean ± S.E.M, * $P < 0.05$, ** $P < 0.01$, Unpaired two-tailed Student's t test.

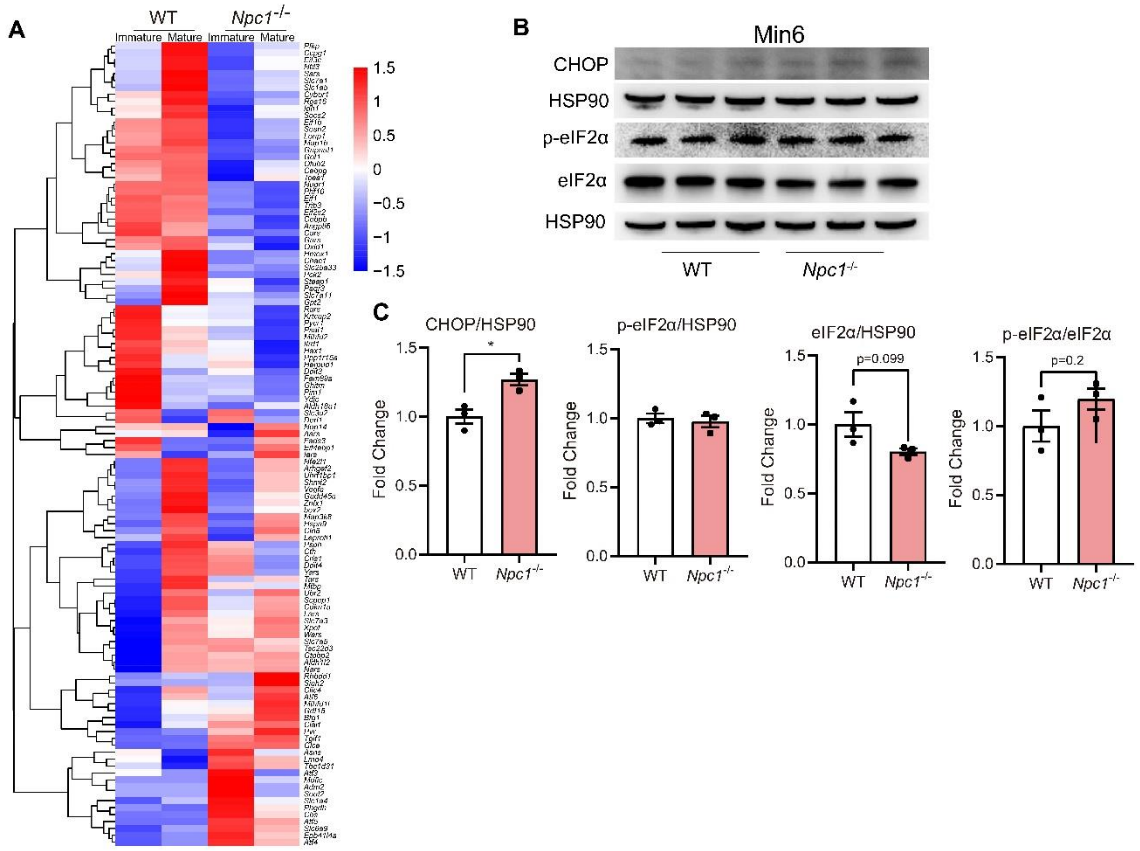

Supplemental Figure 6

A. Heatmap of the average ISR gene expression in scRNA seq data from mature and immature WT and *Npc1*$^{-/-}$ β cells. **B**-**C**. Western blot and quantification of ISR key factors (CHOP, eIF2α, p-eIF2α) in WT and *Npc1*$^{-/-}$ Min6 cells. Data are presented as the mean ± S.E.M, * $P < 0.05$, $P < 0.01$. Unpaired two-tailed Student's t test.

Supplemental Table 1. Primer sequences

Gene	Forward	Reverse
Npc1	*TGTTTGGTATGGAGAGTGTGGA*	*GTCACAGCAGAGACTGACATTG*
Ins1	*CACTTCCTACCCCTGCTGG*	*ACCACAAAGATGCTGTTTGACA*
Ins2	*GCTTCTTCTACACACCCATGTC*	*AGCACTGATCTACAATGCCAC*
Ucn3	*GCTGTGCCCCTCGACCT*	*TGGGCATCAGCATCGCT*
MafA	*TTCAGCAAGGAGGAGGTCAT*	*CTCTGGAGCTGGCACTTCTC*
Pdx1	*CCCCAGTTTACAAGCTCGCT*	*CTCGGTTCCATTCGGGAAAGG*
Slc2a2	*CAGCAGCACTCCACTATGATTG*	*CGTACACCGTCTCCCCTACAA*
Nkx6.1	*TCTGGACAGCAAATCTTCGCCC*	*ACTTGGTCCTGCGGTTCTGGAA*
Slc30a8	*CAGAGAACTTCGACAGAAGCC*	*CTTGCTTGCTCGACCTGTT*
Gck	*TGAGCCGGATGCAGAAGGA*	*GCAACATCTTTACACTGGCCT*
Txnip	*TCTTTTGAGGTGGTCTTCAACG*	*GCTTTGACTCGGGTAACTTCACA*
Ldha	*TGTCTCCAGCAAAGACTACTGT*	*GACTGTACTTGACAATGTTGGGA*
Aldob	*GAAACCGCCTGCAAAGGATAA*	*GAGGGTCTCGTGGAAAAGGAT*
Olfm1	*CACCGAACTCACCCAAGTGTT*	*CACTGTGCAGATACACCTGCC*
Fgf1	*CCCTGACCGAGAGGTTCAAC*	*GTCCCTTGTCCCATCCACG*
Dlk1	*AGTGCGAAACCTGGGTGTC*	*GCCTCCTTGTTGAAAGTGGTCA*
Ngn3	*AGTGCTCAGTTCCAATTCCAC*	*CGGCTTCTTCGCTTTTTGCTG*
Aldh1a3	*GGGTCACACTGGAGCTAGGA*	*CTGGCCTCTTCTTGGCGAA*
Hmgcr	*AGCTTGCCCGAATTGTATGTG*	*TCTGTTGTGAACCATGTGACTTC*
Srebf2	*GCAGCAACGGGACCATTCT*	*CCCCATGACTAAGTCCTTCAACT*
Ldlr	*TGACTCAGACGAACAAGGCTG*	*ATCTAGGCAATCTCGGTCTCC*
Npc2	*AGGACTGCGGCTCTAAGGT*	*AGGCTCAGGAATAGGGAAGGG*
Lamp1	*CAGCACTCTTTGAGGTGAAAAAC*	*ACGATCTGAGAACCATTCGCA*
Lamp2	*TGTATTTGGCTAATGGCTCAGC*	*TATGGGCACAAGGAAGTTGTC*
Ctsd	*GCTTCCGGTCTTTGACAACCT*	*CACCAAGCATTAGTTCTCCTCC*
Ctsb	*TCCTTGATCCTTCTTTCTTGCC*	*ACAGTGCCACACAGCTTCTTC*
Cox6a2	*CTGCTCCCTTAACTGCTGGAT*	*GATTGTGGAAAAGCGTGTGGT*
Uqcrc2	*AAAGTTGCCCCGAAGGTTAAA*	*GAGCATAGTTTTCCAGAGAAGCA*
Uqcrc1	*AGACCCAGGTCAGCATCTTG*	*GCCGATTCTTTGTTCCCTTGA*
Atp5a1	*TCTCCATGCCTCTAACACTCG*	*CCAGGTCAACAGACGTGTCAG*
Atp4a	*GATGGAGATTAACGACCACCAG*	*ACGGGCAAACTTCACATACTC*
Pink1	*TTCTTCCGCCAGTCGGTAG*	*CTGCTTCTCCTCGATCAGCC*
Bnip3	*TCCTGGGTAGAACTGCACTTC*	*GCTGGGCATCCAACAGTATTT*
Clec16a	*AGACCCTTCCTGGATATGGTG*	*GAGGGCGTGATAGTCATCGTC*
Dnm1L	*CAGGAATTGTTACGGTTCCCTAA*	*CCTGAATTAACTTGTCCCGTGA*
Huwe1	*TCTTCCACTAGAGATTCTGCCG*	*TGATACCAGCAAGGGGATCTTC*
Tomm7	*ATCCGCTGGGGCTTTATTCC*	*CGACGGTTCAGGCATTCCA*
Uba52	*GGCCAAGATCCAAGACAAGGA*	*CTGACGAAGGGATGGCTCAA*
Ulk1	*AAGTTCGAGTTCTCTCGCAAG*	*CGATGTTTTCGTGCTTTAGTTCC*
Casp3	*ATGGAGAACAACAAAACCTCAGT*	*TTGCTCCCATGTATGGTCTTTAC*
Actb	*GGCTGTATTCCCCTCCATCG*	*CCAGTTGGTAACAATGCCATGT*
Gapdh	AGGTCGGTGTGAACGGATTTG	TGTAGACCATGTAGTTGAGGTCA
